# EXPLORING THE IMPLEMENTATION OF THE STRENGTHENING A PALLIATIVE APPROACH IN LONG-TERM CARE PROGRAM IN A CLINICAL TRIAL

**DOI:** 10.1093/geroni/igad104.1811

**Published:** 2023-12-21

**Authors:** Sharon Kaasalainen, Paulette Hunter, Genevieve Thompson, Abigail Wickson-Griffiths

**Affiliations:** McMaster University, Hamilton, Ontario, Canada; St. Thomas Moore College, University of Saskatchewan, Saskatoon, Saskatchewan, Canada; University of Manitoba, Winnipeg, Manitoba, Canada; University Of Regina, Regina, Saskatchewan, Canada

## Abstract

Despite the high mortality rates in long-term care (LTC) homes, most do not have a formalized palliative program. Hence, our research team developed the Strengthening a Palliative Approach in Long Term Care (SPA-LTC) program. This presentation will address how the SPA-LTC intervention was designed to be scaled up in a large randomized control trial, related implementation issues and strategies used to address them during the trial. This study used a cross-jurisdictional, effectiveness-implementation type II hybrid cluster design to evaluate the SPA-LTC program in 18 LTC homes across three provinces in Canada. The implementation component was designed as a formative evaluation to explore how to adapt the SPA-LTC program in real time. The SPA-LTC intervention leveraged both internal (palliative champion team) and external supports (palliative consultants) to build capacity within the LTC home to support and prepare residents and families for end-of-life decision making. Due to staff shortages and their inability to attend lengthy education sessions, we held four one-hour education sessions for the palliative champion team that were led by external palliative consultants over three months. Reach of the intervention was compromised as approximately 10% of participating residents died before a palliative care conference could be held. As a result, we prioritized residents who had lower Palliative Performance Scale (PPS) scores when planning a care conference. Clearly, efforts are needed to evaluate both the implementation and effectiveness of complex interventions, such as a palliative program, within a pragmatic approach given the real-life challenges that currently exist in this sector.

